# Anomaly Detection in Discrete Manufacturing Systems by Pattern Relation Table Approaches

**DOI:** 10.3390/s20205766

**Published:** 2020-10-12

**Authors:** Xinmiao Sun, Ruiqi Li, Zhen Yuan

**Affiliations:** 1 School of Automation and Electrical Engineering, University of Science and Technology Beijing, Beijing 100083, China; xmsun@ustb.edu.cn (X.S.); g20193024@xs.ustb.edu.cn (Z.Y.); 2 College of Information Science and Technology, Beijing University of Chemical Technology, Beijing 100029, China

**Keywords:** anomaly detection, discrete manufacturing systems, pattern relation table, centralized algorithm, parallel algorithm, Timed Petri Net

## Abstract

Anomaly detection for discrete manufacturing systems is important in intelligent manufacturing. In this paper, we address the problem of anomaly detection for the discrete manufacturing systems with complicated processes, including parallel processes, loop processes, and/or parallel with nested loop sub-processes. Such systems can generate a series of discrete event data during normal operations. Existing methods that deal with the discrete sequence data may not be efficient for the discrete manufacturing systems or methods that are dealing with manufacturing systems only focus on some specific systems. In this paper, we take the middle way and seek to propose an efficient algorithm by applying only the system structure information. Motivated by the system structure information that the loop processes may result in repeated events, we propose two algorithms—centralized pattern relation table algorithm and parallel pattern relation table algorithm—to build one or multiple relation tables between loop pattern elements and individual events. The effectiveness of the proposed algorithms is tested by two artificial data sets that are generated by Timed Petri Nets. The experimental results show that the proposed algorithms can achieve higher AUC and F1-score, even with smaller sized data set compared to the other algorithms and that the parallel algorithm achieves the highest performance with the smallest data set.

## 1. Introduction

Anomaly detection is a hot topic in various domains, such as manufacturing anomaly detection [1,2], cyber attack detection [3,4,5,6], and crowded scene video anomaly detection [7,8]. Cyber attacks detection typically detects three types of external attacks, i.e., false data injection attack, denial-of-service attack, and confidentiality attack. The crowded scene video anomaly detection seeks to detect abnormal human behaviors from the video source. This paper emphasizes on the anomaly detection of discrete manufacturing systems due to the surge of smart manufacturing and Industry 4.0. The smart manufacturing systems—equipped with mass sensors, fast networks, and sophisticated controllers—seek to make production efficient and reliable, which inevitably becomes increasingly complex. Such complexity makes it very difficult for even experts to fully understand the behaviors of the systems and identify anomalies in a timely and precise manner. On the other hand, smart devices (i.e., sensors, controllers) and wireless networks make the data collection and distribution to computers easier, thus promoting extensive researches on data-based anomaly detection algorithms. Based on the type of data, anomaly detection algorithms can deal with time-series data [9,10,11], discrete data [12,13,14,15], or mixed types of data [16].

This paper seeks to deal with anomaly detection for discrete event data that result from discrete manufacturing systems. Discrete manufacturing systems produce distinct items, such as auto mobiles, smart phones, and air planes, which can be easily identified and counted. Producing one item typically involves multiple operations by different machines. The sequential actions of machines, such as turning on a button and turning off the button, define a series of events, thus generating various discrete event data.

**Figure 1:**
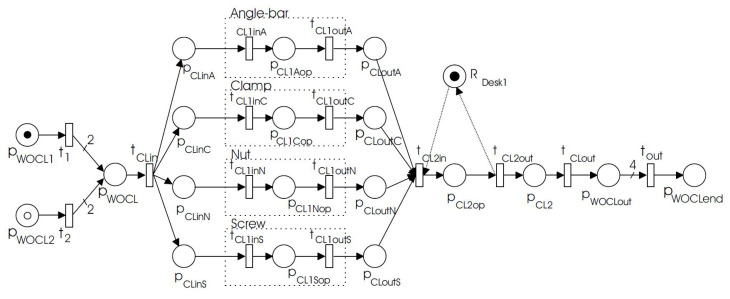
An example of producing an L-shaped corner clamp with four parallel processes (‘Angle-bar with holder L’, ‘Clamp’, ‘Nut’, and ‘Screw’) in Ref. [17].

In the production process, there can be some abnormal activities, besides, the conditions of machines that are involved in the production process might also be abnormal. In this paper, we consider three types of abnormal behaviors: missing necessary events, wrong order of events, and false process injection. Discrete manufacturing systems usually consist of parallel processes to produce different parts of the product that will be assembled later. Figure 1 gives an example of producing an L-shaped corner clamp with four parallel processes, see details in [17]. Besides, the whole process may also include loop processes, e.g., a material will be sent back to the same machine to process again when the material should be re-positioned and re-processed. This paper will take these two features into account when designing the anomaly detection algorithms, and such feature-based algorithms facilitate us to reach good performance at low costs: required small-sized data.

Chandola et. al. reviewed the classical anomaly detection methods in discrete sequences in [12]. The authors summarized four approaches used in sequence-based anomaly detection problems, namely, kernel-based methods, window-based methods, Markovian methods, and Hidden Markov model (HMM)-based methods. Kernel-based methods are one of the similarity-based algorithms, which detect anomalies by comparing the similarity between the testing sequences and the training sequences. These algorithms are tolerant of false negatives, which may result in huge loss when considering the fact that customs may lose their money once ATM has a problem and manufacturing companies may suffer great losses once severe abnormal behaviors occur. Thus, in this paper, we set a strict bar to anomalous behaviors: as long as anomalous factor appears, the testing sequence will be considered to be abnormal. Window-based algorithms first cut the long sequence by windows of length *k* and then compare the similarity of each window. The shortcoming of window-based algorithms is that it is sensitive to the selection of *k*, cannot be optimized easily. The Markovian, HMM, or Automata (see [15]) model-based algorithms focus on modeling the transition of states, which cannot reflect parallel processes, since parallel transitions may result in the same state changes as sequential processes. Recently, machine learning and deep learning based algorithms spring up, such as in [10,18]. It is essentially a model-based algorithm and the model can be described as a certain weighted structure of a network. This usually needs a larger data set to train a network.

The aforementioned algorithms mainly focus on the performance (usually measured by F-score), but pay little attention to how much data are required to reach such performance. Nowadays, although data collection is cheap, data cleaning and processing may also be labor extensive and time-consuming. This paper will evaluate both the algorithm performance and size of the data set required.

In this paper, we proposed a centralized pattern relation-based algorithm (Algorithm 1) and a parallel pattern relation-based algorithm (Algorithm 2) to detect anomalies in manufacturing systems, especially for parallel processes and loop processes. Algorithm 1 seeks to build a relation table between patterns—combinations of events that are meaningful in the models. Algorithm 2 seeks to build multiple relation tables for each pattern. Besides, developed algorithms provide two ways in searching for patterns that can describe the loop processes.

To test the proposed algorithms, we generated two artificial data sets due to the following reasons: (1) in reality, it is expensive or impossible to design an experiment to get large data sets with balanced data. (2) For new products or the products in the design stage, there are limited data. (3) Artificial data sets can easily provide more scenarios to test the robustness of the proposed algorithms. Data sets are generated through simulation techniques of Timed Petri Net that can model parallel and loop processes.

Overall, the contributions of this paper are threefold. First, two novel approaches are proposed in order to perform the anomaly detection. Second, the proposed approaches are tested for two complex manufacturing systems, one is a parallel system with loop processes, and the other one is a parallel system with nested loop processes. Finally, we investigate the size of the data that are needed to achieve a certain level of performance. The simulation results showed that the AUC values of our proposed algorithms can reach up to 0.92 with as little as 50 training sequences and up to 1 with more sequences.

The paper is organized, as follows. Section 2 formulates the anomaly detection problem and overviews two proposed relation-based anomaly detection approaches. Section 3 describes Algorithm 1 and Section 4 describes Algorithm 2 in detail, respectively. In Section 5, we present the simulation results and conclude in Section 6.

## 2. Pattern Relation-Based Anomaly Detection Approaches

### 2.1. Problem Formulation

The anomaly detection problem can be defined as

**Given:** a training data set *D* consists of *N* normal sequences, each of which contains a series of basic events $e_{i}$ (denoted by lower case English letters in the example, which can be extended to the Unicode set).

**Task:** for any new sequence $\sigma_{i}$, detect $\sigma_{i}$ to see whether it is normal or abnormal.

**Example:** with the training data set D={afhk,afgfghjfhjhk,afhgjhfgfgfk}, then for each sequence $\sigma_{i}$ in $D^{\prime }=\left[afgjhfk,afgfhk\right]$, identify each $\sigma_{i}$ as normal or abnormal. This data set will be used to illustrate the proposed algorithms steps-by-steps through the paper.

### 2.2. Overview of the Proposed Algorithms

The main idea of the proposed relation-based anomaly detection algorithms follows such a pipeline: first, train a model with normal data sets, and then do the anomaly detection based on the model. The “core piece” of the model is so-called pattern relation tables, where the pattern is a combination of events that can represent a special relation of the events. The differences between the two algorithms proposed are the way of detecting patterns and building the relation tables.

We first define some common notations that are used in describing the models.

Let $D=\{d_{1},\ldots ,d_{N}\}$ be the training data set, and $d_{i}\left(i\in \left[1,\ldots ,N\right]\right)$ is a sequence containing events occurred in order in a normal process (e.g., $d_{i}$ can be afhk, where each letter represents an event).

Let $E=\{e_{1},\ldots ,e_{K}\}$ be the event set of all the unique events existed in the data set, and *K* is the cardinality of set *E* (e.g., in the Example, E={a,f,h,k,g,j}, and K=|E|=6). Let $E_{n}$ be the necessary event set, which contains the events that appeared in every sequence $d_{i}$ (thus, in the Example, $E_{n}=\left\{a,f,h,k\right\}$). Let $E_{r}$ be the repeat event set, where the repeat event appeared at least twice in a sequence. In the Example, $E_{r}=\left\{f,g,h,j\right\}$.

Let $LP=\{lp_{1},\ldots ,lp_{n}\}$ be the loop pattern set that contains repeatedly appeared sub-sequences with length varies in the range of [2,K] in *D*. In the two proposed methods, LPs are different. Let P=LP∪E be the pattern set and the pattern set should include the elements that can fully recover the training set *D*. Note that the choice of *P* is not unique. For example, the simplest one is LP=∅ and P=E, and the most useless one is LP=D and P=D∩E which doesn’t provide any new information from the training set. How to extract a suitable *P* is the key to the proposed algorithms.

Let *T* be the pattern relation table whose row index and column index equal to elements in *P* (Algorithm 1) or elements in each LP (Algorithm 2). A table cell represents an order relation between the two elements in the sequences. The relation tables are also different in the two proposed algorithms: Algorithm 1 builds one table in a centralized way while Algorithm 2 builds |LP|+1 tables in a parallel way. We will introduce them in detail in each subsection.

**Figure 2:**
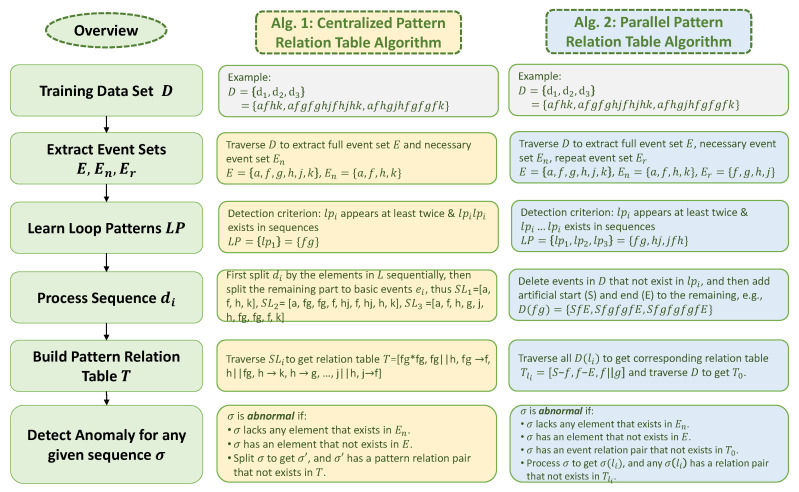
Overview of major steps in relation-based anomaly detection algorithms and introduce the main idea of each step in Algorithms 1 and 2.

Now, we are ready to define the model $M=\{E,E_{n},E_{r},LP,P,T\}$ and we will introduce how we train M from *D* for different algorithms. Both of the algorithms require four steps to learn M and the final step is to do the anomaly detection. Figure 2 provides an overview of the approaches and summarizes the main idea of each step in Algorithms 1 and 2. Note that, not all elements in M will be used in each algorithm. Algorithm 1 needs $\left\{E,E_{n},LP,P,T\right\}$ and Algorithm 2 needs $\left\{E,E_{n},E_{r},LP,T_{0,\ldots ,|LP|}\right\}$.

## 3. Algorithm 1: Centralized Pattern Relation Table Algorithm

Algorithm 1 summaries the five steps in the centralized pattern relation table algorithm and the following subsections elaborate each step.
**Algorithm 1** Centralized Pattern Relation Table Algorithm**Input:** Training data set *D*, test sequence σ**Output:** Detect if σ is *normal* or *abnormal*1:Extract the event set *E* and the necessary event set $E_{n}$.2:Learn the loop pattern set LP by Subroutines 1 and 2.3:Split $d_{i}\in D$ by Subroutine 3.4:Build the Pattern Relation Table *T* by Subroutine 4.5:Detect if σ is normal or not by Subroutine 5.

### 3.1. Extract the Event Sets E and the Necessary Event Set $E_{n}$

Traverse all sequences $d_{i}$ in the training data set *D*, extract the event set $E=\{e_{1},\ldots ,e_{K}\}$ (the cardinality K=|E|) and the necessary event set $E_{n}=\left\{e_{n_{1}},\ldots ,e_{n_{m}}\right\}$. Subsequently, the event set *E* can be decoupled into two subsets: necessary event set $E_{n}$ and the unnecessary event set $E_{u}=E\setminus E_{n}$. In the Example, E={a,f,g,h,j,k}, K=6, $E_{n}=\left\{a,f,h,k\right\}$, and $E_{u}=\left\{g,j\right\}$.

### 3.2. Learn the Loop Pattern Set

We observed that, in practical discrete manufacturing systems, the events that are involved in the loops must be repeated at least twice in the sequence. Accordingly, we define a loop pattern $lp_{i}$ as some sequential events (at least two events) repeatedly appeared in any single sequence $d_{i}$. The learning process of the loop pattern set is implemented by Subroutines 1 and 2.

Subroutine 1 illustrates the process of extracting a loop pattern candidate list *C*. Candidate $c_{i}$ is obtained by traversing all $d_{i}$ to find sub-strings of length 2 to *K* and appearance frequency >1. Besides, the elements in *C* is ordered by its importance value—defined by the length of $c_{i}$ multiply its appearance frequency—in the training data. We use the Example to show the details.

In the Example, there are no sub-strings with length ≥2 appeared for more than once in $d_{1}=afhk$, so no loop pattern candidate can be extracted. Then for $d_{2}=afgfgfhjfhjhk$, we start with the length-of-2 sub-string af, and find its appearance frequency is only 1, so discard it; according to the running basis, we then come to fg, and add it to *C* since its frequency is 2; append gf,hj,jh due to the same reason; go on until the end of $d_{2}$. And then come to traversing the length-of-3 sub-strings, afg appears only once, so discard it, as well as fgf whose frequency is only 1; continue this process until the end. And no patterns with length >3 that appeared more than once. Perform the same process for $d_{3}$. So eventually, the C=[fg,gf,hj]. Meanwhile, calculate the importance value for each element in *C* and reorder *C* by the importance (the importance values of elements in *C* are 8(fg),8(gf),4(hj), respectively. Subsequently, the ordered C=[fg,gf,hj]. It is worth noting that, although af has appeared in all three sequences $d_{i}$ in *D*, yet it does not appear twice or more in any single sequence, so it was not added into *C*.
**Subroutine 1***Candidate Patterns Extracting* (D,|E|)**Input:** Data set *D*, event cardinality *K***Output:** Ordered candidate pattern set *C*1:C←∅,Map=set(seq,importance)2:**for**$d_{i}\in D$**do**3: **for** w← sub-string of $d_{i}$ with $\left|w\right|\in \left[2,\min\{\right|d_{i}\left|,K\}\right]$ **do**4:  num← frequency of *w* in $d_{i}$5:  **if** num>1 **then**
6:   Map.add(*w*,0) **if** w∉Map.keys()7:   importance←importance+len(w)×num
8:  **end if**
9: **end for**
10:**end for**11:Order the Map based on importance in a descending order12:**return**Map.keys()

Note that the candidate *C* may include redundant elements, since, if a longer sub-sequence $c_{i}$ repeated more than twice, then all of the sub-sequences $c_{i}$ will be repeated more than twice and become the candidates automatically. Therefore, we need a eliminate routine to select the most proper loop pattern elements among them. Moreover, a loop pattern element may exist in the form of $c_{i}c_{i}$ in any sequence. Accordingly, the next step is to filter the candidates by Subroutine 2 in order to obtain the true loop pattern set LP. Steps 1–6 in Subroutine 2 is to eliminate latter candidates that can be represented by a former candidate together with some basic elements. Steps 7–14 are to eliminate candidate $c_{i}$ that never appeared with the form $c_{i},c_{i}$ in any sequence $d_{i}$.

Continued the above example, the candidate loop pattern set *C* has three elements fg,gf,hj. We then check the first one (i.e., fg) to see whether it is a subset of any other pattern, if so, then the latter element will be marked as −1 and won’t be added into the loop pattern set LP (in the Example, gf is a subset of fg, so gf will be marked as −1); then, check the second one (gf), and do the same process, and if it is marked as −1, we will skip it and go on. Eventually, We add all of the candidate patterns not marked as −1 to the loop pattern LP. So far, the loop pattern candidate is LP={fg,hj}. Subsequently, test whether there exists the form $lp_{1},lp_{1}$ for the loop element $lp_{i}\in LP$ in the split list SL obtained from splitting $d_{i}$ by the current LP with Subroutine 3.

In the Example, $SL_{1}=\left[a,f,h,k\right],SL_{2}=\left[a,fg,fg,f,hj,f,hj,h,k\right],SL_{3}=\left[a,f,h,g,j,h,fg,fg,f,k\right]$, then if sequential $lp_{1},lp_{1}$ exists in $SL_{i}$, then $lp_{i}$ will be retained; otherwise, it will be discarded. Here, fg,fg exists in $SL_{2}$ and $SL_{3}$, but hj,hj doesn’t exist in any $SL_{i}$, so, eventually, LP={fg}.
**Subroutine 2***Pattern Learning Subroutine* (*C*)**Input:** Ordered candidate pattern set *C***Output:** Loop pattern set LP1:LP←∅2:**for** candidate $c_{i}$!=-1 in *C* **do**3: **for** $c_{j}$ in C[i+1:len(C)] **do**4:  $c_{j}\leftarrow$ -1 **if** $c_{i}\subseteq c_{j}$5: **end for**
6: LP.append($c_{i}$) **if** $c_{i}$!=-17:**end for**8:**for**$d_{i}$ in *D* **do**9: SL← Sequence Processing Subroutine($d_{i},LP,E$)10: **for** $lp_{i}$ in LP **do**11:  **if** no sequential $lp_{i},lp_{i}$ in SL **then**12:   LP.remove($lp_{i}$)13:  **end if**
14: **end for**15:**end for**16:**return**LP

### 3.3. Sequence Processing Subroutine

The Sequence Processing Subroutine (σ,S,E) (Subroutine 3) returns an ordered list SL by splitting any given sequence σ with a given ordered list *S* and the basic event set *E*. This subroutine will be applied in getting the loop pattern set LP and building the relation table *T* later.

This subroutine starts with splitting σ by $s_{i}$ in *S* one by one (so $s_{1}$ is of the highest priority, then $s_{2}$ and so on) in Steps 1–5. For the remaining part that not match any $s_{i}$ will be split by basic events $e_{i}$ in *E*, see Steps 6–12. Note that SL is an ordered list and concatenate all the elements in SL can obtain the original sequence σ. In the Example, if *S* is [fg,hj], then the split of $d_{1}=afhk$ will be $SL_{1}=\left[a,f,h,k\right]$, since there is no sub-sequence includes any element in LP, $d_{2}=afgfgfhjfhjhk$ will be $SL_{2}=[a,fg,fg,f,hj,$f,hj,h,k], and $d_{3}=afhgjhfgfgfk$ will be $SL_{3}=\left[a,f,h,g,j,h,fg,fg,f,k\right]$.
**Subroutine 3***Sequence Processing Subroutine* (σ,S,E)**Input:** Any sequence σ, given ordered list *S* and event set *E***Output:** A split-list SL obtained from splitting σ by *S* and *E* sequentially  $\sigma_{0}\leftarrow \sigma$  SL = ←∅  **for** $s_{i}$ in *S*(i∈[1,|S|]) **do**   $\sigma_{i}\leftarrow$ replace $s_{i}$ by ’-’+$s_{i}$+’-’ in $\sigma_{i-1}$  **end for**  $\sigma_{\left|S\right|}$.replace(’–’,’-’)  **for** each symbol sp in $\sigma_{\left|S\right|}\left[1:-1\right]$.split(’-’) **do**   **if** sp in *S* **then**    SL.append(sp)   **else**    SL.append(sp split by basic events $e_{i}$)   **end if**  **end for**  **return** SL

### 3.4. Build a Relation Table

Define the pattern set P=LP∪E as the union of the loop pattern set and the basic event set. Now, we are ready to build a pattern table *T* to describe the relationship between pattern events for a given data set *D*. There are three types of relations:

- $p_{i}\to p_{j},i\neq j$, meaning that pattern $p_{i}$ appears ahead of $p_{j}$, but $p_{j}$ cannot be ahead of $p_{j}$.
- $p_{i}\|p_{j},i\neq j$, meaning that $p_{i}\to p_{j}$ and $p_{j}\to p_{i}$ both exist in the normal data set.
- $p_{i}*p_{i}$, meaning that this pattern can repeat itself as many times as possible. We can infer that any loop pattern $lp_{i}$ must have this relation.

Subroutine 4 provides more details of building such a relation table and Table 1 shows the result for the data set *D* in the Example.
**Subroutine 4***Pattern Table Building Subroutine* (LP,E,D)**Input:** Loop Pattern LP, Event Set *E*, Training Data Set *D***Output:** Pattern Table *T* Pattern set P←LP∪E, Pattern table $T_{\left|P|\times |P\right|}\leftarrow \emptyset$ **for** sequence $d_{i}$ in *D* **do**  SL = *Sequence Splitting Routine*($d_{i}$, LP, *D*)  **for** ($sl_{i},sl_{i+1}$) in SL **do**   $T(sl_{i},sl_{i+1})=*$**if** $sl_{i}$ equals to $sl_{i+1}$   $T(sl_{i},sl_{i+1})=\to$ **if** $T(sl_{i},sl_{i+1})$ **is empty or →, and** $T(sl_{i+1},sl_{i})$ **is empty**
   $T\left(sl_{i},sl_{i+1}\right),T\left(sl_{i+1},sl_{i}\right)=\|$ **if** $T(sl_{i},sl_{i+1})$ **is empty or →, and** $T(sl_{i+1},sl_{i})=\to$
  **end for**
 **end for**
 **return** *T*


### 3.5. Anomaly Detection Algorithm

Now, the model $M=\{E,E_{n},LP,PT\}$ has been trained from the normal data set *D*. Accordingly, three rules are proposed to detect anomalous behaviors in a new sequence σ:

- σ lacks any element in the necessary event set $E_{n}$.
- σ has any element that is not in the basic event set *E*.
- Split σ by Subroutine 3 to obtain SL, check in *T* and the cell $T(SL_{i},SL_{i+1})$ is empty.

The first rule is to ensure that the normal sequence should have the necessary events in *D*. The second rule is to ensure that there is no event out of the basic event set *E*. For the third one, we believe the normal orders between the pattern elements have existed in *T*. See the details in Subroutine 5.

The centralized algorithm shows all of the relations between pattern elements in one table *T*. Consider that the systems may be complex and the dimension of *T* is large, this algorithm will be time-consuming. Subsequently, we propose a parallel algorithm that allows for multiple such *T*s to be used simultaneously.
**Subroutine 5** Anomaly Detection of Any Sequence σ**Input:** Model $M=\{E,E_{n},LP,P,T\}$, test sequence σ**Output:** Detect if σ is *normal* or *abnormal*1:Extract the event set $\hat{E}$ of σ.2:**if**$\hat{E}⊉E_{n}$ or $E⊉\hat{E}$ **then**3: **return** σ is *abnormal*.4:**end if**5:SL←*Sequence Splitting Routine*(σ,LP,E)6:**for** pattern pair ($p_{i},p_{i+1}$) in SL **do**7: **if** $T(p_{i},p_{i+1})$ is *∅* **then**8:  **return** σ is *abnormal*.9: **end if**
10:**end for**11:**return**σ is *normal*.

## 4. Algorithm 2: Parallel Pattern Relation Table Algorithm

The parallel pattern relation table algorithm in Algorithm 2 has the same work flow as in Algorithm 1. However, in this algorithm, we take a different point of view of the loops in the data set, i.e., when the loop process happens, there must exist some events that will be repeated and the combinations of repeated events show the patterns of the loops. Therefore, a repeated event set needs to be learned. Besides, to extract multiple pattern relation tables in a parallel way, the subroutines to process a sequence and learn the loop pattern set will be changed accordingly. We will introduce the difference in the following subsections.
**Algorithm 2** Parallel Pattern Relation Table Algorithm**Input:** Training data set *D*, test sequence σ**Output:** Detect if σ is *normal* or *abnormal*1:Extract the event set *E* and the necessary event set $E_{n}$.2:Extract the repeated event set $E_{r}$ by Subroutine 6.3:Learn the loop pattern set LP introduced in Section 4.2.4:Process the sequence $d_{i}\in D$ in parallel introduced in Section 4.3.5:Build multiple relation tables in parallel introduced in Section 4.4.6:Detect if σ is normal or not by Subroutine 7.

### 4.1. Extract the Repeated Event

Similar to Algorithm 1, this algorithm also needs to extract the event set *E* and the necessary event set $E_{n}$ from the normal training data set *D*. Subsequently, a repeated event set $E_{r}$ whose elements appear at least twice in a single sequence is extracted. Subroutine 6 provides a way to extract the $E_{r}$. In the Example, $E_{r}=\left\{f,g,h,j\right\}$.
**Subroutine 6***Repeated Events Extracting Subroutine* (D,E)**Input:** Training data set *D*, Event set *E*
**Output:** Repeated event set $E_{r}$1:$E_{r}\leftarrow \emptyset$2:**for** sequence $d_{i}$ in *D* **do**3: **for** event $e_{i}$ in *E* **do**4:  **if** pattern $e_{i}\ldots e_{i}$ in $d_{i}$**and** $e_{i}\notin E_{r}$ **then**5:   $E_{r}$.add($e_{i}$)6:  **end if**
7: **end for**
8:**end for**9:**return**$E_{r}$

### 4.2. Learn the Loop Pattern Set

In this step, first, process the data set *D* get a modified data set $\hat{D}$ by traversing over *D* and removing all events not in $E_{r}$ while keeping the original order. Subsequently, the remaining process can be done in a parallel way. For each element in $E_{r_{1}}$, extract the candidate loop pattern set $C(E_{r_{i}})$ from $\hat{D}$, where each element in $C(E_{r_{i}})$ is started and ended with the same single event $E_{r_{i}}$. Then sort $C(E_{r_{i}})$ according to the length of the elements therein and the shortest one in $C(E_{r_{i}})$ is the pattern of $C(E_{r_{i}})$. In the Example, $\hat{D}=\left\{fh,fgfgfhjfhjh,fhgjhfgfgf\right\}$, C(f)={fgf,fgfgf,…}, C(j)={jfhj}, C(g)={gfg,gjhfg,…}, C(h)={hjfh,hjfhjh,hjh,hgjh}, so far the loop pattern set LP is LP=fgf,gfg,hjh,jfhj. However, for each pattern element, the order of the events is not necessary, just keep the event set of the pattern elements and remove the redundant ones and get the final loop pattern set. Continue the Example, set(gfg)=set(fgf)=fg, so gfg is identified as redundant and it will be removed from LP, eventually, LP=[fg,hj,jfh].

### 4.3. Sequence Processing Subroutine

The third step is to define a subroutine to process any given sequence σ based on each element in LP and obtain a corresponding new sequence $\sigma (LP_{i})$. It is still a removal process that is based on $LP_{i}$-for each σ remove all elements not in $LP_{i}$, and keep the order of remaining elements. Subsequently, apply it to every sequence in the training data *D* and obtain $D\left(LP_{i}\right)=\left\{d_{1}\left(LP_{i}\right),d_{2}\left(LP_{i}\right),d_{3}\left(LP_{i}\right)\right\}$, and add artificial start (S) and end(E) symbols to every $d_{i}\left(LP_{i}\right)$ at its two ends. For example, $LP_{1}=fg$, then $D\left(LP_{1}\right)=D\left(fg\right)=\left\{SfE,SfgfgfE,SfgfgfgfE\right\}$, $D\left(LP_{2}\right)=D\left(hj\right)=\left\{ShE,ShjhjhE,ShjhE\right\}$, and $D\left(LP_{3}\right)=D\left(jfh\right)=\left\{SfhE,SffhjfhjhE,SfhjhfffE\right\}$.

### 4.4. Build Multiple Relation Tables

Afterwards, the fourth step is to build multiple relation tables $T_{i}$ based on $D(LP_{i})$ (then the number of $T_{i}$ equals to |LP|). In addition, we also build a basic event relation table $T_{0}$ (see Table 2) based on consecutive event relation in the original training data set *D*. The process of building the relation table is the same, as shown in Subroutine 4, based on the *D*. Accordingly, for $D(LP_{1})$, the corresponding relation table $T_{fg}$ has indices of [S−f,f−E,f−g,g−f] (see Table 3); for $D(LP_{2})$ and $D(LP_{3})$, the corresponding $T_{hj}$ and $T_{jfh}$ are shown in Table 4 and Table 5, respectively.

### 4.5. Parallel Anomaly Detection Algorithm

In the parallel algorithm, the anomaly detection is based on |LP|+1 relation tables $T_{i}\left(i\in [0,\ldots ,|LP|]\right)$. Only when all of the relation pairs obtained from processing σ are shown to be normal, the sequence will be a normal one; otherwise, it is abnormal. Subroutine 7 summarizes this process.

It is worth noting that after extracting the loop pattern set LP, the algorithm can be run in parallel based on the element in LP to speed up the algorithm. That’s why we called it “Parallel Anomaly Detection Algorithm”.
**Subroutine 7** Parallel Relation Table Anomaly Detection Algorithm**Input:** Model $M=\{E,E_{n},E_{r},LP,T_{0},\ldots ,T_{\left|LP\right|}\}$, test sequence σ**Output:** Detect if σ is normal or not1:Extract the event set $\hat{E}$ of σ.2:**if**$\hat{E}⊉E_{n}$**or**$E⊉\hat{E}$**then**3: **return** σ is *abnormal*.4:**end if**5:**for** event pair ($e_{i},e_{i+1}$) in σ **do**6: **if** $T_{0}\left(e_{i},e_{i+1}\right)$ is *∅* **then**7:  **return** σ is *abnormal*.8: **end if**
9:**end for**10:**for** Loop pattern $lp_{i}$ in LP **do**11: $\sigma (lp_{i})$ = *Sequence processing*($\sigma ,lp_{i}$)12: **for** event pair ($e_{i},e_{i+1}$) in $\sigma (lp_{i})$ **do**13:  **if** $T_{i}\left(e_{i},e_{i+1}\right)$ is *∅* **then**14:   **return** σ is *abnormal*.15:  **end if**
16: **end for**
17:**end for**18:**return**σ is *normal*.

## 5. Simulation

In this section, we test the proposed algorithms with two artificial data sets. The data sets are generated by a discrete event model Timed Petri Nets and we begin with briefly introduce the definitions and mechanisms of the Timed Petri Net. A detailed introduction can be found in the book [18].

### 5.1. Timed Petri Net

Petri Net is a classical model of Discrete Event Systems. When compared with Automata, it is more straightforward to demonstrate discrete manufacturing systems, such as parallel processes and loop processes.

A Timed Petri Net is a six-tuple (P,T,A,w,x,V), where

- *P* is a set of places, that is conditions required to enable transitions.
- *T* is a set of transitions.
- A⊂(P×T)∪(T×P) contains all arcs from places to transitions and from transitions to places.
- w:A→{1,2,3,…} is the weight function on the arcs.
- *x* is the marking function of the Petri Net, where a marking represents the number of tokens in each place in the net.
- $V=\{v_{j}:t_{j}\in T\}$ is a clock structure and the clock sequence $v_{j}$ is associated with a transition $t_{j}$.

The clock structure $v_{j}=\left\{v_{j1},v_{j2},\ldots \right\}$ means that, if the k−1th fired time of the transition $t_{j}$ is *t*, then the next fired time could be $t^{\prime }=t+v_{jk}$.

**Figure 3:**
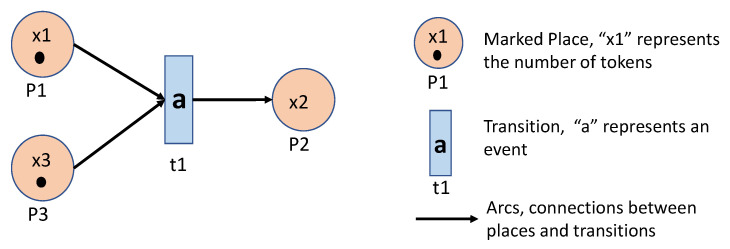
A Petri Net Graph Example.

A Petri net graph can visualize a Petri net. Figure 3 shows a Petri net with $P=\{p_{1},p_{2},p_{3}\}$, $T=\{t_{1}\}$, $A=\{\left(p_{1},t_{1}\right)\left(p_{3},t_{1}\right),\left(t_{1},p_{2}\right)\}$, $w(p_{1},t_{1})=1$, $w(p_{3},t_{1})=1$, $w(t_{1},p_{2})=1$. The marking function x=[1,0,1]. A possible clock structure is $v_{1}=\left\{1,2,1,2,\ldots \right\}$.

A transition $t_{j}\in T$ is defined as enabled or feasible if $x\left(p_{i}\right)\geq w\left(p_{i},t_{j}\right)$ for all $p_{i}\in I\left(t_{j}\right)$, where $I\left(t_{j}\right)=\left\{p_{i}\in P:\left(p_{i},t_{j}\right)\in A\right\}$ contains the input places to transition $t_{j}$. In other words, transition $t_{j}$ is enabled when the number of tokens in $p_{i}$ is at least as large as the weight of the arc connecting $p_{i}$ to $t_{j}$, for all places $p_{i}$ that are inputs to transition $t_{j}$. A transition $t_{j}$ is fired if it happened at some time and then moved tokens to the next places. For example, in Figure 3, we can say $t_{1}$ is enabled during the period [0,1), and fired at t=1.

When a transition is fired, then we can say an event occurred. To obtain a sequence of events, we need the dynamics of the Petri Net, i.e., the way of moving tokens from places to places. It is defined by $x^{{}^{\prime }}\left(p_{i}\right)=x\left(p_{i}\right)-w\left(p_{i},t_{j}\right)+w\left(t_{j},p_{i}\right)$ and one can easily identify it through a Petri Net graph.

Given an initial state $x_{0}$ and a Timed Petri Net, a sequence of fired events can be obtained by repeating the following steps: check the enabled transitions, fired the transition that is of the minimal scheduled time, and changes of states. This is the main idea of generating an event sequence, and a detailed description can be found in Chapter 10 of Ref. [18].

### 5.2. Experimental Systems

**Figure 4:**
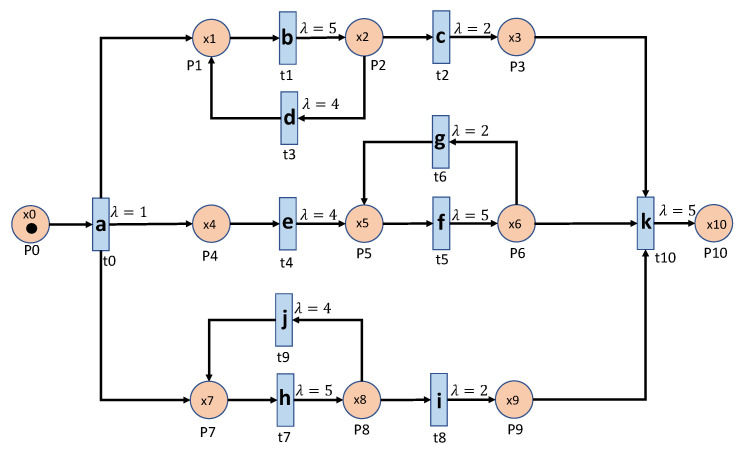
Parallel Loop process with loop events.

**Figure 5:**
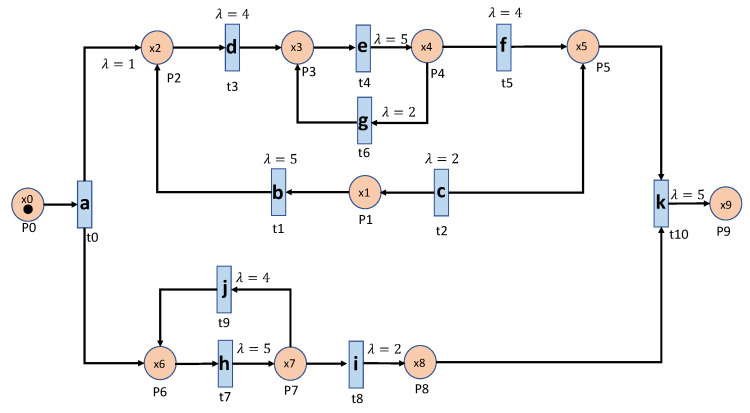
Parallel Loop process with nested loop events.

As we mentioned, we are interested in detecting any anomaly that exists in parallel processes, loop processes, and even nested loops and combinations of all these factors in more complex systems. Besides, the operation time (the interval time between two events) of a machine might be stochastic. Figure 4 and Figure 5 show the Petri Net graphs of two such manufacturing systems. Figure 4 represents a system that consists of three parallel processes and each process has a loop process, which is the system used in [14] for comparison purposes. Figure 5 contains parallel processes and nested loop processes that make the systems more complex. We will show that the proposed algorithms are also capable of dealing with such a system. In both systems, the stochastic operation time is modeled by a Poisson process with parameter λ.

### 5.3. Data Generation

With these two systems and the data generation approaches, we can generate normal data sets. However, in order to test our algorithms, we also need to generate abnormal data sets. We introduce three types of abnormal behaviors, as follows:

- Introduce an erroneous event transition to a normal sequence.
- Remove a necessary event from a normal sequence.
- Introduce some unrecorded/unknown events in a normal sequence.

Each type of anomalies accounts for 1/3 of sequences in the abnormal data set. Assume that the number of sequences in the training data set is *N*, in the testing data set, half of them are normal sequences, and the other half are abnormal ones.

### 5.4. Compared Approaches

We will compare our algorithms with the prefix tree algorithms and the basic event table algorithm, as introduced in [14].

The prefix tree algorithm strictly compares the difference between the normal sequences and test sequence. When the testing sequence differs from any of the normal sequences, it is abnormal. In order to record the normal sequences in less space and to loop them up at high speed, the “prefix tree” data structure is used, hence its name.

The basic event table-based algorithm builds an event relation table between any two consecutive events in a normal sequence. When a testing sequence is given, if any two consecutive events within it do not exist in the relation table, then it is identified as abnormal.

### 5.5. Performance Metrics

Anomaly detection problems can also be viewed as a binary classification problem. Typical metrics for evaluating a classification algorithm include accuracy, sensitivity, precision, F-1 score, and AUC (area under curve). In reality, manufacturing systems can generate much more normal data than the abnormal one, so the data set will be highly unbalanced. We choose the F-1 score and AUC score as the performance metrics, as the accuracy does not work well under such circumstances. The F-1 score is calculated from the precision and recall, namely

(1) $$F-1=2\times \frac{precision\times recall}{precision+recall}$$

where the precision is defined as

(2) $$precision=\frac{\mathrm{true}\;\mathrm{positive}}{\mathrm{true}\;\mathrm{positive}+\mathrm{false}\;\mathrm{positive}}$$

and the recall is defined as

(3) $$recall=\frac{\mathrm{true}\;\mathrm{positive}}{\mathrm{true}\;\mathrm{positive}+\mathrm{false}\;\mathrm{negative}}$$

The AUC is the area under the ROC (Receiver Operating Characteristic) curve, where an ROC curve plots the true positive rate vs. false positive rate at different classification thresholds. AUC represents the probability that a random positive example is positioned to the right of a random negative example. When AUC>0.5, the classification result is better than a random classification method.

Besides, we plot the relationship between the number of sequences (*N*) in the training data set and the AUC and the F-1 score in order to investigate the efficiency of the proposed algorithm.

### 5.6. Simulation Results and Discussion

**Figure 6:**
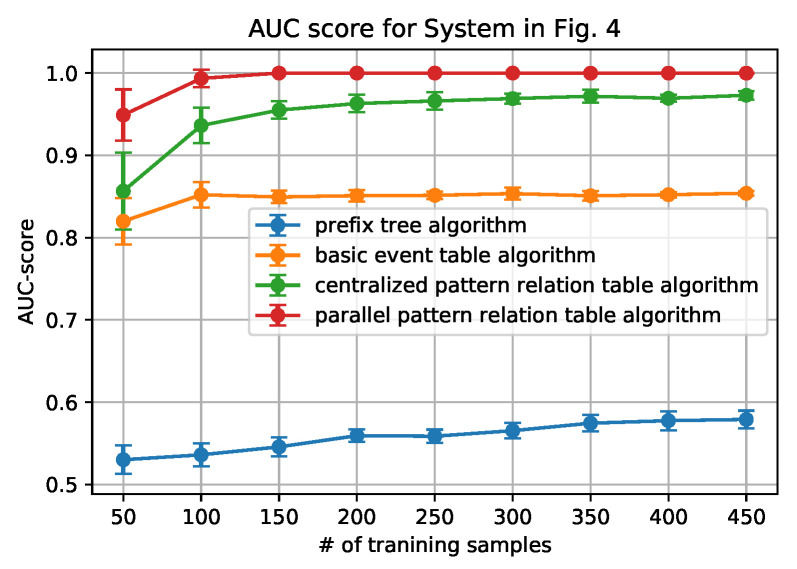
AUC score for System in Figure 4.

**Figure 7:**
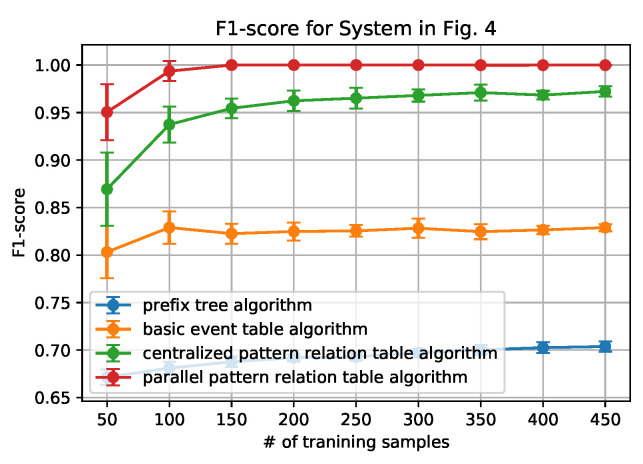
F-1 score for System in Figure 4.

**Figure 8:**
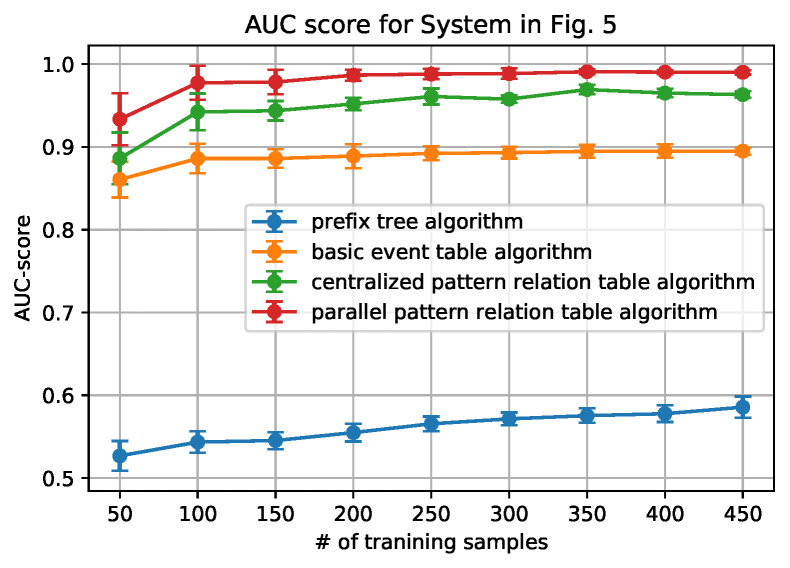
AUC score for System in Figure 5.

**Figure 9:**
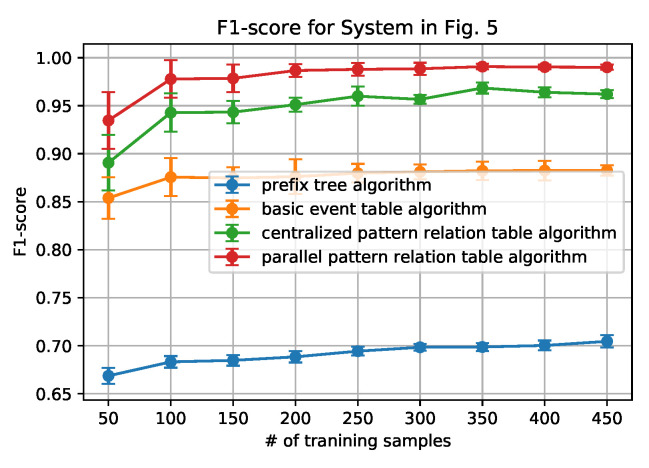
F-1 score for System in Figure 5.

Figure 6, Figure 7, Figure 8 and Figure 9 show how the AUC and F-1 score vary with the number of sequences *N* in the training data sets, where the dots represent the average values, and the upper and lower bars represent the standard deviations. These figures are obtained by the following steps: (1) for each *N*, we ran the data generation routine for 10 times to get 10 data sets, which contain *N* sequences of each set; (2) performed the anomaly detection algorithms; (3) calculated AUC and F-1 Score on each data set, and obtained the average and standard deviation; and, (4) draw the figures.

From the figures, we can conclude that the newly proposed algorithms-parallel pattern relation table algorithm and the centralized one-outperform the prefix tree algorithm and the basic event table algorithm. This is because there are inherent shortages in the latter two algorithms. In the prefix tree algorithm, the criterion of being detected as normal is strict that only the sequences that existed in the training data set can be identified. However, because there are loop processes, the number of normal sequences is unlimited. Therefore given a small size of training data, some actual normal cases (especially the loop ones) cannot be identified, thus making the F1-score and the AUC-score low. With the size of the normal data set grows, the performance will be better, but will not be able to achieve 1.

In the basic event table algorithm, it only considers the event relationship between the consecutive events in a sequence. This might misidentify the cases where some necessary events are missing as normal. Taking the case that is shown in Figure 4 as an example, the sequence ‘abhcefgfk’ and ‘abhcefgik’ are mistakenly identified as normal, since there exist all of the consecutive events, yet ‘abhcefgfk’ misses ‘i’ and ‘abhcefgik’ misses ‘f’ between ‘fg’ and ‘k’.

In the centralized pattern relation table algorithm and the parallel pattern relation algorithm, they are designed to capture the loop patterns by focusing on the loop patterns or the repeated events. In the centralized algorithm, it overcomes the shortage of the basic event table algorithm by checking if all of the necessary events are included in the tested sequence. However, it still fails to detect the cases ‘abhcefgik’, since ‘fg’ is also normal to be followed by ‘i’. To address this issue, the parallel algorithm only focuses on the relationship between the events within the loop patterns by the deletion process of the sequences (sequence processing subroutine); moreover, it also builds the relationship with an artificial start (S) and end (E). For instance, only ‘f’ can be connected to ‘S’ and ‘E’ while ‘g’ cannot, as shown in Table 3. Applying the sequence processing subroutine, ‘abhcefgik’ will be ‘SfgE’ and it is detected as abnormal. That is why it can correctly identify the ‘abhcefgik’ case.

In addition, it is worth noting that the parallel algorithm can achieve higher performance with a smaller size of the data set. This is also owing to the sequence processing subroutine. In the parallel algorithm, as long as repeated events appear, the relationship between the repeated events can be built in the relation table. However, in the centralized algorithm, the repeated events should be first detected as a pattern and then appear repeatedly. Because the latter case is a subset of the former case, the parallel algorithm will be easier to capture the loop patterns that are based on a smaller sized data set.

Finally, we also note that the relation table based algorithms have their own limitations: the performance converges no matter how the number of the training data set grows. The reason is that, if the number of patterns or the number of the repeated event is fixed, the number of the relationship in the pattern relation tables is limited. As long as all of the relationships appear, the table is fully built.

## 6. Conclusions

We investigated anomaly detection problems in complex discrete manufacturing systems and proposed two effective methods for detecting anomalies of wrong orders, missing events, and unrecorded/unknown events. By fully taking the system structure (not the entire model) into account, our algorithms work well, even with quite small-sized training data sets. This implies that our methods can apply to the fields, where only limited data can be obtained and partial structure information is given, such as in the test of prototypes of intelligent sensor/control devices. In reality, such structure information is usually easier to be observed.

Note that, although our proposed algorithms seek to be applied in discrete manufacturing systems, they can also be used to other fields if parallel processes and loop processes exist, such as ATM fraud detection. Besides, the idea of searching for patterns in the centralized pattern relation algorithm may be applied to the natural language learning fields to learn the frequent words and grammar between the words.

Future work could be extended to several directions: (1) anomaly detection for totally unlabeled data, which includes both normal data and abnormal data; and, (2) event duration anomaly detection with normal data or unlabeled data.

## Figures and Tables

**Table 1:** Pattern Relation Table *T* of *D*.

	fg	h	f	a	k	g	j
fg	*	‖	→				
h	‖				→	→	‖
f		→			→		
a	→		→				
k							
g							→
j		‖	→				

**Table 2:** Pattern Relation Table $T_{0}$.

	a	f	h	g	j	k
a		→				
f			‖	‖		→
h		‖			‖	→
g		‖	→	→		
j		→	‖			
k						

**Table 3:** Pattern Relation Table $T_{fg}$.

	S	f	g	E
S		→		
f			‖	→
g		‖		
E				

**Table 4:** Pattern Relation Table $T_{hj}$.

	S	h	j	E
S		→		
h			‖	→
j		‖		
E				

**Table 5:** Pattern Relation Table $T_{jfh}$.

	S	j	f	h	E
S			→		
j			→	‖	
f			‖	‖	→
h		‖	‖		→
E					

## Abbreviations

The following abbreviations are used in this manuscript:

HMM	Hidden Markov model
ATM	Automatic Teller Machine
AUC	Area Under Curve
ROC	Receiver Operating Characteristic
